# Accuracy of individual and combined risk-scale items in the prediction of repetition of self-harm: multicentre prospective cohort study

**DOI:** 10.1192/bjo.2020.123

**Published:** 2020-12-02

**Authors:** Anna Kathryn Taylor, Sarah Steeg, Leah Quinlivan, David Gunnell, Keith Hawton, Nav Kapur

**Affiliations:** Centre for Mental Health and Safety, Manchester Academic Health Science Centre, University of Manchester, UK; Centre for Mental Health and Safety, Manchester Academic Health Science Centre, University of Manchester, UK; Centre for Mental Health and Safety, Manchester Academic Health Science Centre, University of Manchester, UK; and NIHR Greater Manchester Patient Safety Translational Research Centre, University of Manchester, UK; Department of Population Health Sciences, University of Bristol, UK; Centre for Suicide Research University Department of Psychiatry, Warneford Hospital, UK; and Oxford Health NHS Foundation Trust, Warneford Hospital, UK; Centre for Mental Health and Safety, Manchester Academic Health Science Centre, University of Manchester, UK; and NIHR Greater Manchester Patient Safety Translational Research Centre, University of Manchester, UK

**Keywords:** Risk assessment, rating scales, suicide, self-harm, statistical methodology

## Abstract

**Background:**

Individuals attending emergency departments following self-harm have increased risks of future self-harm. Despite the common use of risk scales in self-harm assessment, there is growing evidence that combinations of risk factors do not accurately identify those at greatest risk of further self-harm and suicide.

**Aims:**

To evaluate and compare predictive accuracy in prediction of repeat self-harm from clinician and patient ratings of risk, individual risk-scale items and a scale constructed with top-performing items.

**Method:**

We conducted secondary analysis of data from a five-hospital multicentre prospective cohort study of participants referred to psychiatric liaison services following self-harm. We tested predictive utility of items from five risk scales: Manchester Self-Harm Rule, ReACT Self-Harm Rule, SAD PERSONS, Modified SAD PERSONS, Barratt Impulsiveness Scale and clinician and patient risk estimates. Area under the curve (AUC), sensitivity, specificity, predictive values and likelihood ratios were used to evaluate predictive accuracy, with sensitivity analyses using classification-tree regression.

**Results:**

A total of 483 self-harm episodes were included, and 145 (30%) were followed by a repeat presentation within 6 months. AUC of individual items ranged from 0.43–0.65. Combining best performing items resulted in an AUC of 0.56. Some individual items outperformed the scale they originated from; no items were superior to clinician or patient risk estimations.

**Conclusions:**

No individual or combination of items outperformed patients’ or clinicians’ ratings. This suggests there are limitations to combining risk factors to predict risk of self-harm repetition. Risk scales should have little role in the management of people who have self-harmed.

## Background

The third annual progress report of England's National Suicide Prevention Strategy highlighted self-harm as a key issue in its own right, including the need to recognise that people who present to hospital following self-harm are a high-risk group for later suicide.^1^ Emergency departments in England treat more than 220 000 episodes of self-harm annually.^2^ At least half of people who die by suicide have a history of self-harm.^3^ Furthermore, in England self-harm is associated with a 50 times greater risk of suicide in the year after the episode, which may be higher for those who present repeatedly.^4–6^

Hospital presentations involving self-harm can place significant pressure on emergency and mental health services, but provide an opportunity for suicide prevention.^7^ The National Institute for Health and Care Excellence have set out pathways for short- and longer-term assessment and management of self-harm, emphasising the importance of psychosocial assessment by a mental health specialist for every presentation involving self-harm. The risk of further self-harm and specific follow-up care based on the needs of individuals should be considered as part of this assessment.^8,9^

## Use of tools to assess risk

The use of risk scales as part of an assessment following self-harm is widespread. Over 20 tools were found to be used in 32 hospitals in England.^10^ However, their use is controversial; some clinical guidance advises the use of risk scales over locally developed proformas, but others argue that scales should only be used to structure assessments and not to predict future risk of suicidal behaviour or decide upon aftercare.^9^ There is growing evidence that risk scales do not accurately predict repeat self-harm and suicide.^11–14^ In a prospective cohort study of patients referred to liaison psychiatry following self-harm in England, clinician and patient evaluations outperformed risk scales in predicting risk of repeat self-harm.^11^ In a large study of 4000 patients presenting to emergency departments following self-harm, Steeg et al^14^ found risk scales failed to accurately predict both repeat self-harm and suicide. Other research into the use of risk scales in more specific populations (for example children and young people, adults with autism) also found a lack of evidence supporting the use of risk scales in predicting suicide attempts.^15,16^ Recent meta-analyses have suggested that there is no reliable means of distinguishing individuals at high risk from those at low risk of suicidal behaviour; diagnostic accuracy of individual risk factors is frequently only slightly better than chance, and using multiple risk factors is not significantly more useful than single factors.^17,18^

Head-to-head comparisons of individual items from psychometrically tested risk scales have not been studied in real-world settings. It is not known how individual risk-scale items perform compared with patient and clinician estimates, and whether a scale constructed using the highest performing items would improve predictive accuracy for repeat self-harm.

## Aims

Building on our previous studies, the aim of this study was to compare the predictive accuracy of individual items from widely used risk scales with clinician and patient estimates of risk of repeat self-harm within 6 months. We also aimed to construct a new scale from items with the highest predictive accuracy in each scale. Using data from a large prospective cohort evaluating risk scales following self-harm,^11^ we hypothesised that some individual scale items would perform better than the overall scale. We also hypothesised that the scale constructed using the best performing items would improve the predictive accuracy for repeat self-harm.

Our specific objectives were to:

- estimate the predictive accuracy of the individual items from risk scales (the Manchester Self-Harm Rule, ReACT Self-Harm Rule, SAD PERSONS Scale, Modified SAD PERSONS Scale, and Barratt Impulsiveness Scale) to determine if any individual scale items had better diagnostic accuracy in predicting repeat self-harm compared with the overall scale and clinician and patient global scales; and
- evaluate and compare the predictive accuracy of a scale constructed from the highest performing individual items from each scale using a range of dual and global diagnostic accuracy performance indicators and a sensitivity analysis using the classification-tree method.

## Method

### Data sources

We used data from a multicentre prospective cohort study that examined the diagnostic accuracy for predicting repeat self-harm of five risk scales: the Manchester Self-Harm Rule,^19^ ReACT Self-Harm Rule,^20^ SAD PERSONS Scale,^21^ Modified SAD PERSONS Scale^22^ and Barratt Impulsiveness Scale.^23^ For full details of the inclusion and exclusion criteria, service provision, case definitions and procedure, see Quinlivan et al.^11^ In summary, participants were patients aged 18 years or over referred between March 2014 and January 2015 from emergency departments in five large teaching hospitals in England to liaison psychiatry services for assessment following self-harm. Each consecutive episode of self-harm, including those by the same individual, was considered as an index episode, reflecting the reality of presentation to emergency services.^19^ The sample size of 480 was determined by a power calculation to enable meaningful comparative differences in predictive accuracy to be detected.^11^ Self-harm was defined as intentional non-fatal self-injury or self-poisoning, irrespective of motivation or degree of suicidal intent.^24^

### Risk scales

The risk scales were previously tested for the predictive accuracy for repeat self-harm and/or attempted suicide.^11,25^ The items in the scales and cut-off points are summarised in the Appendix. The Manchester Self-Harm Rule^19^ and the ReACT Self-Harm Rule^20^ are four-item scales, where the presence of any one or more of the items identifies the patient as high risk. The SAD PERSONS Scale,^21,26^ and Modified SAD PERSONS Scale^22^ categorise patients as low, medium or high risk (Appendix).

There was some overlap in factors in the SAD PERSONS and Modified SAD PERSONS; we therefore combined these to ensure all the factors were included without any repetition. The Barratt Impulsiveness Scale^23,27^ was designed to measure the construct of impulsiveness and includes 30 items rated on a scale of one to four, ranging from rarely/never to almost always/always. Several scale items (items: 9, 20, 1, 7, 8, 12, 13, 10 15 and 29) are reverse scored (for example ‘plan things carefully’). The ordinal data were recoded as binary data, for consistency with the other binary scales. Scale items with a score of three or four were coded as one. Reverse scored items were coded as zero if the score was one or two.

We also included the clinician and patient global evaluation of risk scale.^11^ These global scales consist of a single question that asks the respondent to estimate the likelihood of repeat self-harm within 6 months on a 1–10 Likert-type scale (for example: ‘How likely do you think it is, that [you]/[the patient] will repeat self-harm within the next six months? Please indicate on this scale (with 1 as extremely unlikely and 10 extremely likely)’). We used the midpoint as our cut-off point (i.e. 1–5, low risk; ≥6 high risk). The cut-off point of 5–6 was selected as the optimal threshold for both the patient and clinician global scale using Youden's J index. This was reported in a previous study using the same data.^11^

Risk scales were fully completed, with the exception of the Barratt Impulsiveness Scale where some items had missing data. However, all items had at least 92% complete data. Episodes with missing scale-item data were excluded listwise for analyses relating to that scale item.

### Reference standard

The outcome for the study was hospital-treated repeat self-harm within 6 months of presentation, which was ascertained from self-harm monitoring systems and hospital records of the participating hospitals.^11^ This time frame was selected because this is a high-risk period during which the majority of repeat episodes occur^28^ and has been used as an outcome measure in previous studies.^29^

### Ethical approval

The authors assert that all procedures contributing to this work comply with the ethical standards of the relevant national and institutional committees on human experimentation and with the Helsinki Declaration of 1975, as revised in 2008. All procedures involving human patients were approved by the Central Manchester Research Ethics Committee (REC no: 13/NW/0838). Informed consent was gained from all participants.

### Data analysis

#### Area under the curve

To enable comparisons of the global accuracy of individual scale items, we constructed receiver operating characteristic curves for each scale item and estimated area under the curve (AUC), a global indicator of diagnostic accuracy.^30^ Higher values of AUC indicate greater discriminatory power; an AUC of 1.0 indicates a perfect test and 0.5 indicates that the result is no better than chance.^31^

#### Scale construction

The items with the highest AUC values within each scale were combined in a new scale. The items were combined to generate a new five-item scale, with each factor given equal weighting. The cut-off point with the optimal AUC was determined by maximising the product of the sensitivity and specificity values of the new scale.^32^ The Liu approach maximises overall predictive ability of the scale without prespecifying the prioritisation of sensitivity or specificity; we utilised this because predictive accuracy of the scale items included in the analysis ranged from high sensitivity (and low specificity) to high specificity (low sensitivity). The scale was derived from the entire sample and its predictive accuracy was tested on a 50% random sample of participants in order to validate the predictive performance of the scale.

#### Scale and item comparisons

The diagnostic accuracy of individual items was compared with the other items within each scale, the overall scale, and with the clinician and patient estimations of risk using AUC and 95% CIs. Scale items were also evaluated using a range of dual diagnostic accuracy statistics and 95% CIs, including sensitivity, specificity, positive and negative predictive values and positive and negative likelihood ratios.

### Sensitivity analysis

Classification and regression tree (CART) analysis was used in a sensitivity analysis to test the robustness of our approach. CART analysis uses recursive partitioning methods to split data based on binary variables. A decision tree was created, with the goal of classifying patients into low-risk and high-risk groups.^33^ In this approach, all scale items from the Manchester Self-Harm Rule, ReACT, SAD PERSONS, Modified SAD PERSONS and Barratt Impulsiveness Scale were pooled and the optimal binary splits that classified episodes into risk categories was sought. Optimal splits were obtained using Gini splitting criteria. We did not impose any misclassification costs such as prioritising correct classification of low or high risk. No pruning or stopping rules were used.

Data analysis was conducted in Stata and SPSS.^34^ We followed the STARD guidance (Standards for the Reporting of Diagnostic Accuracy Studies).^35^

## Results

### Demographic characteristics

The cohort study included 483 episodes of self-harm presenting to the five study hospitals, with 12 individuals presenting more than once (prior to follow-up). A total of 30% (145/483) of self-harm episodes were followed by a repeat presentation within 6 months. Of the episodes, 298/483 (61.7%) were in women and 455/483 (94.2%) were in patients from a White ethnic group. The median age was 33 years (interquartile range: 22–42 years, range: 18–88). In total, 359 of the 483 episodes (74.3%) were in individuals who had a self-reported lifetime history of self-harm. Almost two-thirds of the patients had a prior psychiatric history (found in 310/483 episodes, 64.2%). The most common methods of self-harm were self-poisoning (393/483, 81.4%) and 71/483 self-cutting (14.7%). The full clinical and demographic profiles are reported elsewhere.^11^

### Scale item performance: AUC

**Fig. 1:**
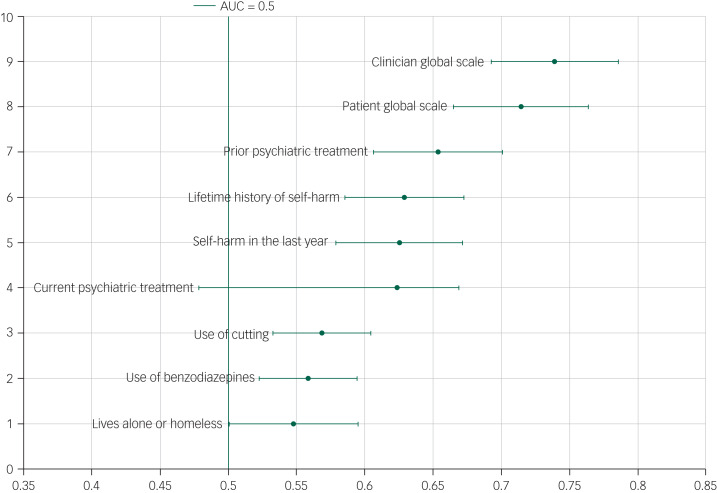
Area under the curve (AUC) and 95% confidence intervals for the Manchester Self-Harm Rule, ReACT rule and the patient and clinician global scales.

**Fig. 2:**
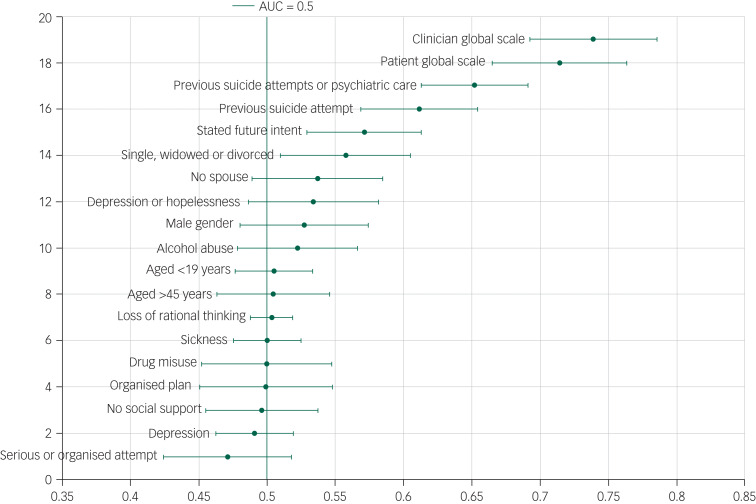
Area under the curve (AUC) and 95% confidence intervals for the SAD PERSONS/Modified SAD PERSONS scales and the patient and clinician global scales.

**Fig. 3:**
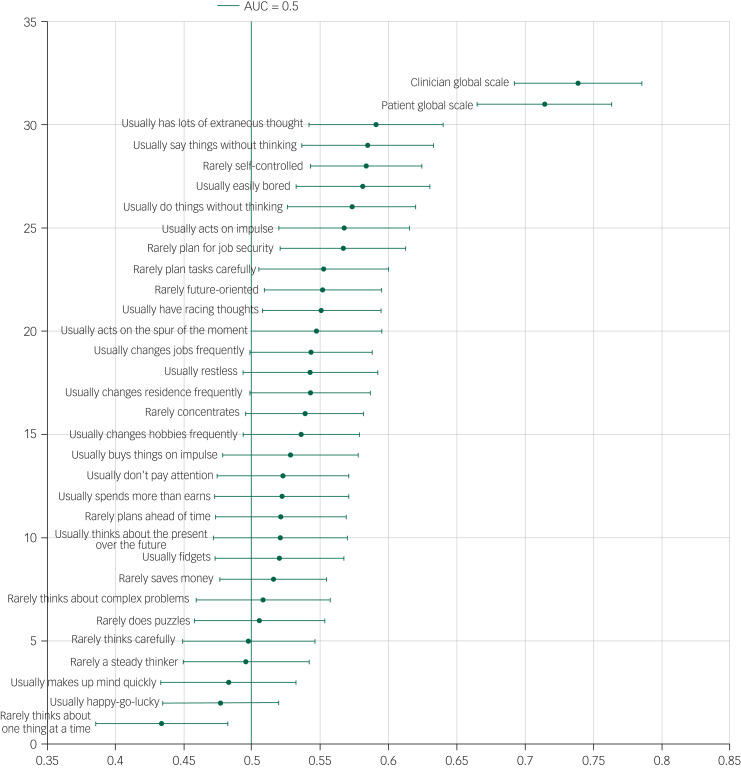
Area under the curve (AUC) and 95% confidence intervals for the Barratt Impulsiveness Scale and the patient and clinician global scales.

The AUC of individual items varied significantly (Figs. 1–3). The item with the lowest AUC (0.43, 95% CI 0.39–0.48) was the Barratt Impulsiveness Scale item ‘rarely thinks about one thing at a time’. The highest AUC (0.65, 95% CI 0.61–0.69) was ‘prior psychiatric treatment’ from the Manchester Self-Harm Rule. Accuracy for the clinician and patient global assessments was better, with AUCs of 0.74 (95% CI 0.69–0.79) and 0.71 (95% CI 0.67–0.76), respectively. The proportions of patients repeating self-harm by scale items are presented in Table 1.

**Table 1:** Repetition of self-harm within 6 months by risk-scale item (total episodes *n* = 483, repeated self-harm *n* = 145, 30%)

Scale items	Data available, *n*	Total episodes with item present, *n* (%)	Repeat self-harm with item present, *n* (%)
Manchester Self-Harm Rule			
Lifetime history of self-harm	483	359 (74)	134 (37)
Prior psychiatric treatment	483	309 (64)	124 (40)
Manchester Self-Harm Rule and ReACT Self-Harm Rule			
Use of benzodiazepines	483	57 (12)	29 (51)
Current psychiatric treatment	483	266 (55)	105 (40)
ReACT Self-Harm Rule			
Self-harm in the last year	483	245 (51)	99 (40)
Use of cutting	483	110 (23)	47 (43)
Lives alone or homeless	483	164 (34)	59 (34)
SAD PERSONS Scale			
Male gender	483	185 (38)	50 (27)
Aged under 19 years	483	43 (8.9)	14 (33)
Aged over 45 years	483	110 (23)	34 (31)
Depression	483	226 (47)	66 (29)
Depression or hopelessness	483	280 (58)	91 (33)
Alcohol abuse	483	128 (27)	43 (34)
Drug abuse	483	50 (10)	15 (30)
Loss of rational thinking	483	11 (2.3)	4 (36)
Single, widowed or divorced	483	264 (55)	91 (35)
Previous suicide attempt	483	304 (63)	114 (38)
Serious or organized attempt	483	36 (7.5)	5 (14)
No social support	483	79 (16)	23 (29)
Organized plan	483	27 (5.6)	8 (30)
Stated future intent	483	95 (20)	43 (45)
No spouse	483	268 (55)	88 (33)
Sickness	483	33 (6.8)	10 (30)
Previous suicide attempts or psychiatric care	483	310 (64)	124 (40)
Barratt Impulsiveness Scale			
Rarely plan tasks carefully	481	273 (57)	93 (34)
Usually do things without thinking	478	273 (57)	97 (36)
Usually make up mind quickly	470	241 (51)	69 (29)
Usually happy-go-lucky	470	124 (26)	33 (27)
Usually don't pay attention	478	185 (39)	60 (32)
Usually have racing thoughts	471	326 (69)	107 (33)
Rarely plan ahead of time	474	282 (59)	90 (32)
Rarely self-controlled	471	332 (70)	116 (35)
Rarely concentrate	476	336 (71)	108 (32)
Rarely save money	474	376 (79)	115 (31)
Usually fidget	459	142 (31)	46 (32)
Rarely think carefully	475	277 (58)	83 (30)
Rarely plan for job security	459	293 (64)	101 (34)
Usually say things without thinking	474	239 (50)	88 (37)
Rarely think about complex problems	470	254 (54)	79 (31)
Usually changes jobs frequently	451	107 (24)	40 (37)
Usually acts on impulse	468	263 (56)	92 (35)
Usually easily bored	471	210 (45)	79 (38)
Usually acts on the spur of the moment	476	269 (57)	91 (34)
Rarely a steady thinker	471	321 (68)	96 (30)
Usually changes residence frequently	465	106 (23)	39 (37)
Usually buys things on impulse	470	238 (51)	76 (32)
Rarely thinks about one thing at a time	479	292 (61)	74 (25)
Usually changes hobbies frequently	467	102 (22)	38 (37)
Usually spends more than earns	470	209 (44)	68 (33)
Usually has lots of extraneous thought	444	233 (52)	88 (38)
Usually thinks about the present over the future	475	255 (54)	81 (32)
Usually restless	468	229 (49)	78 (34)
Rarely does puzzles	475	295 (62)	90 (31)
Rarely future-oriented	474	330 (70)	110 (33)
Clinician Global Scale ≥6	483	228 (47)	107 (47)
Patient Global Scale ≥6	483	226 (47)	101 (45)

Items from the Barratt Impulsiveness Scale were recoded from ordinal to binary to allow comparison with the other binary scales. Scale items with a score of three or four were coded as one. Reverse scored items were coded as zero if the score was one or two.

The following scale items performed better than the scale they originated from: previous suicide attempt (AUC 0.61, 95% CI 0.57–0.65) and previous psychiatric care (AUC 0.65, 95% CI 0.61–0.69) from the SAD PERSONS Scale, which had an overall AUC of 0.55 (95% CI 0.50–0.61) and previous suicide attempt or psychiatric care (AUC 0.65, 95% CI 0.61–0.69) from the Modified SAD PERSONS Scale (overall AUC 0.58, 95% CI 0.53–0.64).

### Scale construction results

The item with the highest AUC in each scale was selected and these were combined to construct a secondary scale. These items were: ‘prior psychiatric treatment’ from the Manchester Self-Harm Rule (AUC 0.65, 95% CI 0.61–0.69), ‘self-harm in the last year’ from the ReACT (AUC 0.63, 95% CI 0.58–0.67), ‘previous suicide attempts’ or ‘psychiatric care’ from the Modified SAD PERSONS (0.65 95% CI 0.61–0.69), and ‘lots of extraneous thought’ from the Barratt Impulsiveness Scale (0.59 95% CI 0.54–0.64). Because of the overlap of the items from the Modified SAD PERSONS and the Manchester Self-Harm Rule, the item from the SAD PERSONS/Modified SAD PERSONS with the next highest AUC was selected (‘stated future intent’, AUC 0.57, 95% CI 0.53–0.61). The cut-off point for the new scale was 0 *v.* ≥1.

The overall sensitivity of the newly constructed scale was 92.7% (95% CI 83.7–97.6%) and specificity was 21.3% (95% CI 15.4–28.1%), the positive predictive value was 31.5% (95% CI 25.1–38.4%), and the negative predictive value was 88.1% (95% CI 74.4–96.0%). The AUC was 0.56 (95% CI 0.52–0.60). The results from the validation sample were similar (AUC 0.56, 95% CI 0.52–0.60). These AUCs were poor in comparison with those for clinician and patient global assessment, which were 0.74 and 0.71, respectively.

### Sensitivity analyses

Results from the CART analysis, which used pooled items from each of the five psychometrically tested scales, suggested the optimal split of the data was by using a single variable, ‘prior psychiatric treatment’ from the Manchester Self-Harm Rule. This resulted in an AUC of 0.65 (95% CI 0.61–0.69), sensitivity of 85.5% (96% CI 78.7–90.8%) and specificity of 45.3% (95% CI 39.9–50.7%). There was no combination of scale items that performed better than this individual item.

### Dual diagnostic accuracy statistics

In terms of each scale, the highest performing item varied depending on which diagnostic accuracy statistic was used (see supplementary Table 1 available online at https://doi.org/10.1192/bjo.2020.123). For the Manchester Self-Harm Rule, ‘lifetime history of self-harm’ had the highest sensitivity (92.4%, 95% CI 86.6–96.2%) but the lowest specificity (33.4%, 95% CI 28.4–38.7%). ‘Use of benzodiazepines’ was most specific (91.7%, 95% CI 88.3–94.4%) but had the lowest sensitivity (20.0%, 95% CI 13.8–27.4%). In the ReACT scale, the use of cutting as a method of harm was the most specific item (81.4%, 95% CI 76.8–85.4%) and the least sensitive (32.5%, 95% CI 24.5–40.7%). In SAD PERSONS, previous suicide attempts or psychiatric care had the highest sensitivity (85.5%, 95% CI 78.7–90.8%), but had relatively poor specificity (45.0%, 95% CI 39.6–50.5%). By contrast, the most specific item (‘loss of rational thinking’, 97.9%, 95% CI 95.8–99.2%) was the least sensitive (2.8%, 95% CI 0.8–6.9%). Finally, on the Barratt Impulsiveness Scale, the most sensitive item was ‘rarely self-controlled’ (82.3%, 95% CI 75.0–88.2% with a specificity of 34.6%, 95% CI 29.4–40.0%), and the most specific was ‘usually changes hobbies frequently’ (80.4%, 95% CI 75.6–84.5%, sensitivity 27.0%, 95% CI 19.8–35.1%).

## Discussion

### Main findings

Our results indicate that individual items from risk scales performed relatively poorly in terms of predicting repeat self-harm. The individual scale items, including history of self-harm, previous psychiatric treatment and suicidal intent failed to outperform clinician and patient global estimations of risk. Scale items relating to previous suicide attempt and previous psychiatric treatment performed slightly better than the SAD PERSONS and Modified SAD PERSONS scales they were part of. Other scale items performed about the same or worse than the overall scales. The scale that we constructed using a combination of the items with the highest predictive accuracy did not enhance the predictive performance for repeat self-harm. Global accuracy was generally poor for the newly constructed scale and did not outperform patient and clinician estimations of risk. Across the individual scale items and constructed scale, more highly sensitive items often had poor specificity, and vice versa. Our findings suggest that despite the potential importance of these items as part of assessments, there is little clinical utility in the use of the items in the prediction of self-harm, either in isolation or combination.

### Strengths and limitations

The patients in our study are similar to patient samples in prior representative multicentre studies in terms of gender, method of self-harm and age.^19,36^ However, patients who did not complete the research assessments or who did not meet eligibility criteria (for example, those who did not speak English, those who were too medically unstable, or those who were actively psychotic) were not included, which could affect generalisability to other populations. The cohort we analysed had a high proportion of participants with a prior history of self-harm (74%) and a high repetition rate (30%), which could suggest that our participants had higher levels of clinical need.^11^

We used multiple methods to test predictive accuracy of scale items used in real-world clinical settings, including the use of algorithmic methods. However, it is possible that alternative machine learning approaches using larger samples would result in improved predictive accuracy. The sample size in the present study was too small to enable prediction of suicide to be assessed. A recent study using routine clinical data, collected as part of a self-harm monitoring system, was sufficiently powered to examine the accuracy of risk scales for predicting suicide.^14^ The four tools measured (Manchester Self-Harm Rule, ReACT, SAD PERSONS and Modified SAD PERSONS) were found to predict suicide deaths less accurately than repeat self-harm episodes. Suicide is a comparatively rare outcome and even greater accuracy is needed in order for predictive tools to be clinically useful.^37^

Whereas two of the scales (Manchester Self-Harm Rule and ReACT) were developed to be used in self-harm risk assessment, the Barratt Impulsiveness Scale is a scale that is not validated specifically as a risk scale for patients who have presented with self-harm (it is a measure of impulsiveness), and the SAD PERSONS Scale was initially developed as a training tool. However, a systematic review conducted prior to the cohort study found that the SAD PERSONS Scale and the Barratt Impulsiveness Scale were being used to predict future suicidal behaviour or repeat self-harm.^10,25,38^

Our methodological approach was based on our primary objectives that were to compare the predictive accuracy of psychometrically tested scale items and combine the top-performing items from each scale. However, the use of a simple additive scale, giving each factor the same weight, may underestimate the predictive accuracy if a key risk factor, such as previous self-harm, is considered equally to an item with lower predictive ability.

We used CART modelling as a sensitivity analysis to test the robustness of our approach and ensure the performance of the new scale was accurately estimated. Although we could have used CART analysis as the primary analysis, this would not have taken into account the risk scale that each item was extracted from, which is how they are used clinically. Logistic regression could have allowed us to select items based on the strength of association between each scale item and the outcome of repeat self-harm. However, we focused on selecting items based on predictive accuracy as the scales are already psychometrically developed, tested and used in clinical practice. Additionally, we categorised the ordinal data in the Barratt Impulsiveness Scale as categorical data, which may result in a loss of information and potential overlap between the groups because of the statistically imposed cut-off. However, recoding was necessary in order to combine the Barratt Impulsiveness Scale items with the binary scale items. Finally, validating the findings on a larger, population-level sample would provide greater accuracy of our findings.

### Comparison with previous research

The previous study by Quinlivan et al^11^ found that the diagnostic accuracy of the full risk scales was not sufficient for them to be of clinical use when used by mental health professionals, and that clinician or global assessment of risk had greater predictive ability for repeat self-harm. A more recent study by Steeg et al^14^ found that utilising risk scales to predict repeat self-harm and future suicide following presentation to hospital is also unsuitable for episodes where mental health professionals are not involved in patient management. A meta-analysis of risk scales used for predicting suicidal behaviour also found that risk scales were not sufficient to be used to predict repeat self-harm and thus treatment allocation.^12^ Although that study examined the individual components of risk scales rather than the risk scales as a whole, our results are similar and suggest that even though some individual items performed slightly better than the scale they originated from, when combined they did not perform better than patient or clinician assessment of risk.

Machine learning methods may present new opportunities for risk prediction.^37,39–41^ Machine learning has been used in several prospective studies on the diagnosis of physical health conditions^42^ including prediction of cancer survival outcomes.^43^ Internationally there is an increasing drive to develop risk scales using machine learning techniques such as random forest, decision trees and support vector machines on ‘big data’.^44^ Machine learning techniques offer some advantages for analysing non-linear observations and large numbers of variables. However, there are a number of potential limitations to using machine learning techniques in mental health settings. When predictive accuracy is prioritised over clinical interpretation, risk prediction models can become highly complex, limiting their clinical utility.^41,45^ Complex algorithms may also shift the focus to prediction and away from clinicians formulating a management plan based on patients’ individual needs and circumstances.^41^

A recent systematic scoping review of the use of machine learning in mental health found that most research is focused on detection, such as natural language processing techniques to detect suicidal ideation from therapy transcripts, and short-term diagnosis rather than on predicting long-term outcomes.^46^ Furthermore, studies did not have a prospective design. One study found that machine learning models could correctly predict a sufficient proportion of suicides among soldiers for the prediction model to have implications for targeting interventions; however, the study focused on a specific population and would not be generalisable to the wider population.^47^ Furthermore, the study did not compare the prediction models to clinician judgement. Other studies using machine learning techniques have been conducted in highly select samples with limited generalisability.^48^ A recent systematic review^49^ found that although suicide prediction models produce overall accurate classification models, their accuracy of predicting a future event is near zero. If machine learning approaches can overcome these limitations, they may result in greater clinical utility. However, our results indicate that no combination or individual items from widely used tested scales outperformed patient and clinician estimations of risk when predicting real-world patient outcomes which suggests there may be limitations to the prediction of suicidal behaviour.

### Clinical implications

This study adds to the growing evidence that risk scales, including components of risk scales, are not suitable for predicting repeat episodes of self-harm or future suicide. The overall performance of all items as measured by the AUC did not exceed a ‘fair’ level of prediction (defined as between 0.7 and 0.8) and none of the items exceeded clinicians’ global assessments of risk for repeat self-harm.^11^ Measures of global accuracy were used to enable head-to-head comparison of scale items. However, measures of dual accuracy highlighted the limitations of scale items for accurately identifying those who repeated and accuracy for those who did not. This has implications for their utility. For example, although some items, such as the highly sensitive ‘lifetime history of self-harm’ item from the Manchester Self-Harm Rule, could be used as a guide for either emergency department or mental health clinicians in their own assessments, the high sensitivity items had poor specificity (and vice versa) so should not be used alone to identify patients as high or low risk and thus affect their treatment allocation. Identifying patients as high risk simply on the basis of meeting one criterion with high sensitivity but poor specificity could result in significant challenges to service provision if risk scales were used to determine patient management because of a potentially large number of referrals to more intensive services.

Factors such as prior psychiatric treatment and lifetime history of self-harm have been previously identified as risk factors for repeat self-harm, however, some Barratt Impulsiveness Scale items such as ‘rarely self-controlled’ and ‘usually changes jobs frequently’ were more specific or sensitive than the other items (although none were both sensitive and specific). Impulsivity and aggression have been correlated with self-harm in a recent systematic review,^50^ so awareness that impulsive/aggressive behaviours may increase the risk of future self-harm may help clinicians assess needs.

Risk classification scales remain in widespread use despite the evidence of their poor predictive abilities.^10,12^ This may be because they act as prompts for factors to consider in formulating a management plan, or because the risk scale results in a score that can be easily interpreted and can instruct the clinician on the management plan (for example, high risk requiring more intensive follow-up). This may be reassuring for less experienced clinicians but is unlikely to be beneficial to their practice or to patients. Furthermore, for relatively rare outcomes such as suicide, classification into high and low risk is unlikely to be clinically useful because of the relatively low positive predictive values that can be obtained. Carter et al^12^ suggest that modifiable risk factors, such as isolation or improving physical health, should be a focus for improvement as part of a holistic assessment of the patient. In addition, patients value a therapeutic alliance,^51^ so focusing on forging a positive and encouraging relationship, as part of a good-quality assessment, may itself reduce the risk of a repeat self-harm episode.^52,53^

The items we measured in the present study were not able to capture the quality and nature of the clinical encounter, which may also contribute to likelihood of repeat self-harm.^51^ Identifying those who are at particularly high risk is challenging; therefore safety planning should be a priority for all patients.^54^ Instead of relying on risk scales to instruct clinicians on the most appropriate management, clinicians should receive comprehensive training and ongoing supervision to improve their knowledge and confidence in assessing patients who self-harm. Research into ‘de-implementation’ of the use of risk scales is an important next step in improving clinical practice for people who have self-harmed. Alternative strategies such as comprehensive patient-centred assessment and safety planning should be explored as part of this.

Given their range of predictive utilities, individual items of scales may be useful for clinicians to consider in their assessments, but they should not be used alone to predict repeat self-harm or determine patient management. Constructing new scales from higher-performing items did not significantly improved performance. The large number of items included in this new scale construction highlights potential limitations to predicting suicidal behaviour accurately.

## Data Availability

Due to the nature of this research, study participants did not agree for their data to be shared publicly and only aggregated data are published.

## Supplementary material

For supplementary material accompanying this paper visit http://dx.doi.org/10.1192/bjo.2020.123. click here to view supplementary material

## Appendix

### Risk scales, their individual items and cut-off points

**Table:**

Risk scale	Scale items	Cut-off points
Manchester Self-Harm Rule^19^	History of self-harm Prior psychiatric treatment Benzodiazepines used as part of the self-harm episode Current psychiatric treatment	Presence of any one item indicates moderate/high risk
ReACT Self-Harm Rule^20^	Recent self-harm (past year) Cutting used as the self-harm method Lives alone or homeless Current psychiatric treatment	Presence of any one item indicates moderate/high risk
The SAD PERSONS Scale^21,26^	Male gender Older age Depression Previous suicide attempt Excess alcohol or substance use Rational thinking loss Social supports lacking Organised plan No spouse Sickness	Three categories of risk: 0–4, 5–6, 7–10 for low, moderate and high, respectively
The Modified SAD PERSONS Scale^22,26^	Male gender Age >19 < 45 years Depression or hopelessness Previous suicide attempt or psychiatric care Rational thinking loss Single, widowed or divorced Organised or serious attempt Social supports lacking Excess alcohol or substance use Stated future intent	Three categories of risk, 0–5, 6–8, 9–14, for low, moderate and high, respectively
The Barratt Impulsiveness Scale^23,27^	30 items based on personality, with responses scored on a 4-point Likert scale	Higher scores indicate greater impulsivity
Patient global estimation of risk^11^	1–10 Likert scale evaluating likelihood of risk of repeat self-harm in the next 6 months	Midpoint (≥6) used to indicate higher risk
Clinician global estimation of risk^11^	1–10 Likert scale evaluating likelihood of risk of repeat self-harm in the next 6 months	Midpoint (≥6) used to indicate higher risk
