# Supplementary material for: Accuracy of individual and combined risk-scale items in the prediction of repetition of self-harm: multicentre prospective cohort study
Source: BJPsych Open. 2020 Dec 2;7(1):e2. doi: 10.1192/bjo.2020.123 (PMC7791570; doi:10.1192/bjo.2020.123)
Supplement: Supplementary file 1 [file bjosup.zip › S2056472420001234sup001.docx]

**Supplementary Table 1** Diagnostic accuracy statistics for 6-month repeat self-harm by risk-scale item

| Subscale questions | Sensitivity, % (95% CI) | Specificity, % (95% CI) | Positive predictive value, % (95% CI) | Negative predictive value, % (95% CI) | Positive likelihood ratio (95% CI) | Negative likelihood ratio (95% CI) | Diagnostic odds ratio (95% CI) |
| --- | --- | --- | --- | --- | --- | --- | --- |
| Lifetime history of self-harm (MSHR) | 92 (87–s96) | 33 (28–39) | 37 (32–43) | 91 (85–95) | 1.4 (1.4–1.4) | 0.2 (0.2–0.3) | 6.1 (3.2–11.8) |
| Prior psychiatric treatment (MSHR) | 86 (79–91) | 45 (40–51) | 40 (35–46) | 88 (82–92) | 1.6 (1.5–1.6) | 0.3 (0.3–0.4) | 4.9 (2.9–8.1) |
| Use of benzodiazepines (MSHR) | 20 (14–27) | 92 (88–94) | 51 (37–64) | 73 (68–77) | 2.4 (1.7–3.4) | 0.9 (0.9–0.9) | 2.8 (1.6–4.9) |
| Current psychiatric treatment (MSHR and ReACT) | 72 (64–80) | 52 (47–58) | 39 (34–46) | 82 (76–86) | 1.5 (1.5–1.6) | 0.5 (0.5–0.6) | 2.9 (1.9–4.4) |
| Self-harm in the last year (ReACT) | 68 (60–76) | 57 (51–62) | 40 (34–47) | 81 (75–85) | 1.6 (1.6–1.6) | 0.6 (0.5–0.6) | 2.8 (1.9–4.3) |
| Use of cutting (ReACT) | 32 (25–41) | 81 (77–85) | 43 (33–53) | 74 (69–78) | 1.7 (1.6–2.0) | 0.8 (0.8–0.9) | 2.1 (1.4–3.3) |
| Lives alone or homeless (ReACT) | 41 (33–49) | 69 (64–74) | 36 (29–44) | 73 (68–78) | 1.3 (1.2–1.4) | 0.9 (0.8–0.9) | 1.5 (1.0–2.3) |
| Male gender (SADPERSONS) | 34 (27–43) | 60 (55–65) | 27 (21–34) | 68 (63–73) | 0.9 (0.8–0.9) | 1.1 (1.1–1.1) | 0.8 (0.5–1.2) |
| Aged under 19 y (SADPERSONS) | 9.7 (5.4–15.7) | 91 (88–94) | 33 (19–49) | 70 (66–74) | 1.1 (0.3–4.5) | 1.0 (1.0–1.0) | 1.1 (0.6–2.2) |
| Aged over 45 y (SADPERSONS) | 23 (17–31) | 78 (73–82) | 31 (22–40) | 70 (65–75) | 1.0 (0.8–1.3) | 1.0 (1.0–1.0) | 1.0 (0.7–1.7) |
| Depression (SADPERSONS) | 46 (37–54) | 53 (47–58) | 29 (23–36) | 69 (63–75) | 1.0 (0.9–1.0) | 1.0 (1.0–1.1) | 0.9 (0.6–1.4) |
| Depression or hopelessness (SADPERSONS) | 63 (54–71) | 44 (39–50) | 33 (27–38) | 73 (67–79) | 1.1 (1.1–1.2) | 0.8 (0.8–0.9) | 1.3 (0.9–2.0) |
| Alcohol abuse (SADPERSONS) | 30 (22–38) | 75 (70–79) | 34 (25–42) | 71 (66–76) | 1.2 (1.0–1.4) | 0.9 (0.9–1.0) | 1.3 (0.8–1.9) |
| Drug abuse (SADPERSONS) | 10 (5.9–16) | 90 (86–93) | 30 (18–45) | 70 (65–74) | 1.0 (0.3–3.3) | 1.0 (1.0–1.0) | 1.0 (0.5–1.9) |
| Loss of rational thinking (SADPERSONS) | 2.8 (0.8–6.9) | 98 (96–99) | 36 (11–69) | 70 (66–74) | 1.0 (0.0–55910000) | 1.0 (1.0–1.0) | 1.3 (0.4–4.7) |
| Single, widowed or divorced (SADPERSONS) | 63 (54–71) | 49 (44–54) | 34 (29–41) | 75 (69–81) | 1.2 (1.2–1.2) | 0.8 (0.7–0.8) | 1.6 (1.1–2.4) |
| Previous suicide attempt (SADPERSONS) | 79 (71–85) | 44 (38–49) | 38 (32–43) | 83 (76–88) | 1.4 (1.4–1.4) | 0.5 (0.5–0.5) | 2.9 (1.8–4.5) |
| Serious or organized attempt (SADPERSONS) | 3.5 (1.1–7.9) | 91 (87–94) | 14 (4.7–30) | 69 (64–73) | 0.4 (0.0– 23410) | 1.1 (1.1–1.1) | 0.4 (0.1–1.0) |
| No social support (SADPERSONS) | 16 (10–23) | 83 (79–87) | 29 (19–40) | 70 (65–74) | 1.0 (0.6–1.6) | 1.0 (1.0–1.0) | 0.9 (0.6–1.6) |
| Organized plan (SADPERSONS) | 5.5 (2.4–11) | 94 (91–97) | 30 (14–50) | 70 (66–74) | 1.0 (0.0–72) | 1.0 (1.0–1.0) | 1.0 (0.4–2.3) |
| Stated future intent (SADPERSONS) | 30 (22–38) | 85 (80–88) | 45 (35–56) | 74 (69–78) | 1.9 (1.7–2.2) | 0.8 (0.8–0.9) | 2.3 (1.5–3.7) |
| No spouse (SADPERSONS) | 61 (52–69) | 47 (41–52) | 33 (27–39) | 73 (67–79) | 1.1 (1.1–1.2) | 0.8 (0.8–0.9) | 1.4 (0.9–2.0) |
| Sickness (SADPERSONS) | 6.9 (3.4–12) | 93 (90–96) | 30 (16–49) | 70 (66–74) | 1.0 (0.1–16) | 1.0 (1.0–1.0) | 1.0 (0.5–2.2) |
| Previous suicide attempts or psychiatric care (SADPERSONS) | 86 (79–91) | 45 (40–50) | 40 (35–46) | 88 (82–92) | 1.6 (1.5–1.6) | 0.3 (0.3–0.4) | 4.8 (2.9–8.0) |
| Rarely plan tasks carefully (BIS) | 64 (56–72) | 46 (41–52) | 34 (28–40) | 75 (69–81) | 1.2 (1.2–1.2) | 0.8 (0.7–0.8) | 1.6 (1.0–1.3) |
| Usually do things without thinking (BIS) | 67 (59–75) | 47 (42–53) | 36 (30–42) | 77 (71–83) | 1.3 (1.3–1.3) | 0.7 (0.7–0.7) | 1.9 (1.2–2.8) |
| Usually make up mind quickly (BIS) | 49 (40–57) | 48 (42–53) | 29 (23–35) | 69 (62–75) | 0.9 (0.9–1.0) | 1.1 (1.0–1.1) | 0.9 (0.6–1.3) |
| Usually happy-go-lucky (BIS) | 23 (17–31) | 72 (67–77) | 27 (19–35) | 69 (63–73) | 0.8 (0.7–1.0) | 1.1 (1.0–1.1) | 0.8 (0.5–1.3) |
| Usually don’t pay attention (BIS) | 42 (34–50) | 63 (57–68) | 32 (26–40) | 72 (66–77) | 1.1 (1.1–1.2) | 0.9 (0.9–1.0) | 1.2 (0.8–1.8) |
| Usually have racing thoughts (BIS) | 76 (69–83) | 34 (29–39) | 33 (28–38) | 77 (70–84) | 1.2 (1.1–1.2) | 0.7 (0.6–0.7) | 1.7 (1.1–2.6) |
| Rarely plan ahead of time (BIS) | 63 (54–70) | 42 (36–47) | 32 (27–38) | 72 (65–78) | 1.1 (1.1–1.1) | 0.9 (0.9–1.0) | 1.2 (0.8–1.8) |
| Rarely self-controlled (BIS) | 82 (75–88) | 35 (29–40) | 35 (30–40) | 82 (75–88) | 1.3 (1.2–1.3) | 0.5 (0.5–0.6) | 2.5 (1.5–4.0) |
| Rarely concentrate (BIS) | 76 (68–83) | 32 (27–37) | 32 (27–37) | 76 (68–83) | 1.1 (1.1–1.1) | 0.8 (0.7–0.8) | 1.5 (0.9–2.3) |
| Rarely save money (BIS) | 82 (74–88) | 22 (17–26) | 31 (26–36) | 73 (64–82) | 1.0 (1.0–1.1) | 0.9 (0.7–1.0) | 1.2 (0.7–2.0) |
| Usually fidget (BIS) | 34 (26–42) | 70 (65–75) | 32 (25–41) | 72 (66–77) | 1.1 (1.0–1.3) | 0.9 (0.9–1.0) | 1.2 (0.8–1.9) |
| Rarely think carefully (BIS) | 58 (50–66) | 42 (36–47) | 30 (25–36) | 69 (62–76) | 1.0 (1.0–1.0) | 1.0 (1.0–1.1) | 1.0 (0.7–1.5) |
| Rarely plan for job security (BIS) | 73 (65–80) | 40 (35–46) | 34 (29–40) | 78 (71–84) | 1.2 (1.2–1.3) | 0.7 (0.6–0.7) | 1.8 (1.2–2.8) |
| Usually say things without thinking (BIS) | 62 (54–70) | 55 (49–60) | 37 (31–43) | 77 (72–83) | 1.4 (1.3–1.4) | 0.7 (0.7–0.7) | 2.0 (1.3–3.0) |
| Rarely think about complex problems (BIS) | 55 (47–64) | 46 (41–52) | 31 (25–37) | 70 (64–76) | 1.0 (1.0–1.1) | 1.0 (0.9–1.0) | 1.1 (0.7–1.6) |
| Usually changes jobs frequently (BIS) | 30 (22–38) | 79 (74–83) | 37 (28–47) | 73 (68–77) | 1.4 (1.2–1.6) | 0.9 (0.9–0.9) | 1.6 (1.0–2.5) |
| Usually acts on impulse (BIS) | 66 (57–74) | 48 (42–53) | 35 (29–41) | 77 (70–82) | 1.3 (1.2–1.3) | 0.7 (0.7–0.8) | 1.8 (1.2–2.7) |
| Usually easily bored (BIS) | 56 (47–64) | 60 (55–66) | 38 (31–45) | 76 (71–81) | 1.4 (1.4–1.5) | 0.7 (0.7–0.8) | 1.9 (1.3–2.9) |
| Usually acts on the spur of the moment (BIS) | 63 (55–71) | 46 (41–52) | 34 (28–40) | 74 (68–80) | 1.2 (1.2–1.2) | 0.8 (0.8–0.8) | 1.5 (1.0–2.2) |
| Rarely a steady thinker (BIS) | 68 (59–75) | 32 (27–37) | 30 (25–35) | 69 (61–77) | 1.0 (1.0–1.0) | 1.0 (0.9–1.1) | 1.0 (0.6–1.5) |
| Usually changes residence frequently (BIS) | 29 (21–37) | 80 (75–84) | 37 (28–47) | 73 (68–78) | 1.4 (1.2–1.7) | 0.9 (0.9–0.9) | 1.6 (1.0–2.5) |
| Usually buys things on impulse (BIS) | 55 (46–63) | 51 (46–57) | 32 (26–38) | 73 (67–79) | 1.1 (1.1–1.2) | 0.9 (0.9–0.9) | 1.3 (0.9–1.9) |
| Rarely thinks about one thing at a time (BIS) | 52 (43–60) | 35 (30–40) | 25 (20–31) | 63 (56–70) | 0.8 (0.8–0.8) | 1.4 (1.3–1.5) | 0.6 (0.4–0.9) |
| Usually changes hobbies frequently (BIS) | 27 (20–35) | 80 (76–85) | 37 (28–47) | 72 (67–76) | 1.4 (1.2–1.6) | 0.9 (0.9–0.9) | 1.5 (1.0–2.4) |
| Usually spends more than earns (BIS) | 48 (39–56) | 57 (51–62) | 33 (26–39) | 71 (65–77) | 1.1 (1.1–1.2) | 0.9 (0.9–1.0) | 1.2 (0.8–1.8) |
| Usually has lots of extraneous thought (BIS) | 65 (57–73) | 53 (47–59) | 38 (32–44) | 78 (72–83) | 1.4 (1.4–1.4) | 0.7 (0.6–0.7) | 2.1 (1.4–3.2) |
| Usually thinks about the present over the future (BIS) | 57 (48–65) | 48 (42–53) | 32 (26–38) | 72 (65–78) | 1.1 (1.1–1.1) | 0.9 (0.9–1.0) | 1.2 (0.8–1.8) |
| Usually restless (BIS) | 55 (46–63) | 54 (48–59) | 34 (28–41) | 73 (67–79) | 1.2 (1.2–1.2) | 0.8 (0.8–0.9) | 1.4 (1.0–2.1) |
| Rarely does puzzles (BIS) | 63 (54–71) | 38 (33–44) | 31 (25–36) | 71 (63–77) | 1.0 (1.0–1.0) | 1.0 (0.9–1.0) | 1.0 (0.7–1.6) |
| Rarely future-oriented (BIS) | 77 (69–84) | 34 (28–39) | 33 (28–39) | 77 (69–84) | 1.2 (1.1–1.2) | 0.7 (0.6–0.8) | 1.7 (1.1–2.6) |
| Clinician Global Scale | 74 (66–80) | 64 (59–69) | 47 (41–53) | 85 (80–90) | 2.1 (2.0–2.1) | 0.4 (0.4–0.4) | 5.0 (3.2–7.8) |
| Patient Global Scale | 69 (61–77) | 63 (57–68) | 44 (37–50) | 83 (78–87) | 1.9 (1.8–1.9) | 0.5 (0.5–0.5) | 3.9 (3.3–5.9) |
