# Supplementary material for: Accuracy of individual and combined risk-scale items in the prediction of repetition of self-harm: multicentre prospective cohort study
Source: BJPsych Open. 2020 Dec 2;7(1):e2. doi: 10.1192/bjo.2020.123 (PMC7791570; doi:10.1192/bjo.2020.123)
Supplement: Supplementary file 1 [file bjosup.zip › S2056472420001234sup002.zip!/coi_disclosure_DG.pdf]

Please wait...

If this message is not eventually replaced by the proper contents of the document, your PDF viewer may not be able to display this type of document.

You can upgrade to the latest version of Adobe Reader for Windows®, Mac, or Linux® by visiting http://www.adobe.com/go/reader_download.

For more assistance with Adobe Reader visit http://www.adobe.com/go/acrreader.

Windows is either a registered trademark or a trademark of Microsoft Corporation in the United States and/or other countries. Mac is a trademark of Apple Inc., registered in the United States and other countries. Linux is the registered trademark of Linus Torvalds in the U.S. and other countries.
